# Synergistic Effect of UiO-66 Directly Grown on Kombucha-Derived Bacterial Cellulose for Dye Removal

**DOI:** 10.3390/molecules29133057

**Published:** 2024-06-27

**Authors:** Pierre Plaza-Joly, Arthur Gallois, Florence Bosc-Rouessac, Martin Drobek, Anne Julbe

**Affiliations:** Institut Européen des Membranes (IEM), CNRS, ENSCM, Univ Montpellier, Place Eugène Bataillon, 34095 Montpellier, France; pierre.plaza-joly@umontpellier.fr (P.P.-J.); arthur.gallois@umontpellier.fr (A.G.); martin.drobek@umontpellier.fr (M.D.); anne.julbe@umontpellier.fr (A.J.)

**Keywords:** natural cellulose, MOF, composite filter, dye adsorption, water treatment

## Abstract

Metal–Organic Frameworks (MOFs) are particularly attractive sorbents with great potential for the removal of toxic dye pollutants from industrial wastewaters. The uniform dispersion of MOF particles on suitable substrates then represents a key condition to improve their processability and provide good accessibility to the active sites. In this work, we investigate the efficiency of a natural bacterial cellulose material derived from Kombucha (KBC) as an active functional support for growing and anchoring MOF particles with UiO-66 structures. An original hierarchical microstructure was obtained for the as-developed Kombucha cellulose/UiO-66 (KBC-UiO) composite material, with small MOF crystals (~100 nm) covering the cellulose fibers. Promising adsorption properties were demonstrated for anionic organic dyes such as fluorescein or bromophenol blue in water at pH 5 and pH 7 (more than 90% and 50% removal efficiency, respectively, after 10 min in static conditions). This performance was attributed to both the high accessibility and uniform dispersion of the MOF nanocrystals on the KBC fibers together with the synergistic effects involving the attractive adsorbing properties of UiO-66 and the surface chemistry of KBC. The results of this study provide a simple and generic approach for the design of bio-sourced adsorbents and filters for pollutants abatement and wastewater treatment.

## 1. Introduction

Water contamination represents a major global environmental problem caused by industrial, domestic and environmental influences. As a result, the need for more efficient and cost-effective sanitation technologies is growing [1]. Among all the methods developed for water and wastewater remediation, the adsorption processes have gained tremendous importance, with a variety of adsorbing materials offering relevant physical and chemical properties.

The dye pollutants in industrial wastewater can cause significant health issues if they are not efficiently removed. Metal–Organic Frameworks (MOFs) are particularly attractive sorbents for the abatement of toxic pollutants due to their large specific surface areas, ultra-high porosity, controlled pore sizes, tunable surface chemistry, abundant active adsorption sites and strong host–guest interactions. Compared to more ordinary adsorbents such as zeolites or activated carbons, MOFs are considered to be more versatile materials. Indeed, their structures and physicochemical properties can be easily modulated by selecting different components from a virtually infinite number of metal clusters and organic ligands [2].

Various complex interactions exist between the external surfaces of MOFs and dye molecules (e.g., electrostatic interactions, aromatic cycle π–π stacking interactions, hydrogen bonding, etc.). Most of the reported MOFs are microporous materials regarding their pore widths (access windows < 2 nm). As a result, a great majority of dye molecules are too large to penetrate the internal microporous networks of MOFs and are, thus, preferentially adsorbed on their external surfaces. Consequently, the external surface properties of MOFs and their accessibility exert a great influence on the adsorption efficiency of dye molecules [3].

It is recognized that a support is an essential element for improving the performances of functional materials such as adsorbents. Its main role is to provide a good accessibility to the active sites and, at the same time, prevent the aggregation of small-sized particles, thus providing a guarantee of high activity over time. Various types of supports, organic or inorganic, have been used to deposit/grow MOF particles on their surfaces. The incorporation of MOF particles can be operated via covalent or non-covalent grafting on the support surface (modified or not). Although this approach is simple, versatile and robust, the direct growth of MOF particles on the support is often a preferred strategy for limiting particles’ aggregation and controlling their dispersion. Scaffolds with intricate structures [4] or fibrous networks [5] are typically well adapted to provide both a high surface area and accessibility to the active material. A large variety of fibrous materials have been used to grow/anchor MOF particles (e.g., carbon [6], glass [7], ceramics [8,9], metals [10], polypropylene [11], polyacrylonitrile [6], cellulose [12,13,14] and other textile fibers [5]).

Numerous methodologies (one-pot or multistep) have been developed to control the growth and location of MOF particles on the support surface. The surface of the support could be eventually modified with functional groups (e.g., carboxylate, amino, hydroxo) able to facilitate the grafting of metal species or organic ligands and the subsequent nucleation/growth of MOF crystals [15,16]. Polyaniline and polydopamine have been proven efficient as mediating surface layers favoring MOF growth [17,18]. In other cases, the reactive seeding strategy is preferred to facilitate the growth of uniform MOF layers, whatever the type of fiber. This method involves the deposition of a thin layer of metal or metal oxide (e.g., ZnO [6], Al_2_O_3_ [6], Co [19], TiO_2_ [11], etc.) and its subsequent conversion to a MOF structure through reaction with the adequate reactants/ligands.

Among the different types of supports, cellulose is an attractive bio-sourced material that has been largely investigated for the preparation of cellulose–MOF composites [12,13,14]. They are used as an adsorbent for the abatement of organic pollutants such as iodine, volatile organic compounds (VOCs), drugs, antibiotics, endocrine disruptors, pesticides, toxins, phenolic compounds and a large variety of dyes [12].

The presence of functional groups such as carboxyl and hydroxyl in cellulose favors the interactions with the positive charges of metals [20]. For example, zinc ions tend to form complexes with the hemiacetal oxygen atom and hydroxyl groups’ anhydroglucose unit of cellulose [21]. Such interactions are important for the in situ synthesis of MOFs into cellulose via the addition of metal salts, followed by organic linkers and solvothermal or hydrothermal treatment. Although these conditions may damage the cellulose and MOF loading is hardly controlled, in situ synthesis remains a simple procedure for obtaining strong interactions between MOFs and cellulose fibers. This approach might also result in faster MOF growth and generate a homogeneous distribution of MOF crystals, with a controlled morphology and possibly smaller crystal size [12]. Moreover, the addition of metals or organic linkers may lead to changes in the conformational structure of cellulose [22]. Whatever the geometry of the cellulosic support (e.g., foams, aerogels, filter papers), it is important to ensure that the synthesized MOFs are not deeply embedded into the cellulose fibers in order to allow the fast diffusion of reactants or adsorbates to the MOF crystals. Such a design warrants high adsorption efficiencies, short contact times and good recyclability.

Due to its excellent structural and functional properties [23,24], bacterial cellulose (BC) is an attractive biopolymer, recently revealed as a promising support for MOF growth and application in several areas [23]. BC can be made via different methods, which are usually simple, eco-friendly and inexpensive [25]. The symbiotic culture of bacteria and yeast (SCOBY), typically used in the fermentation of Kombucha tea, is a bacterial cellulose commonly considered to be a discarded by-product. In addition to its biodegradability and biocompatibility, the Kombucha-derived BC (KBC) exhibits a relatively high degree of crystallinity (from ~30% for the pristine SCOBY up to >80% after chemical cleaning [26,27]) and attractive mechanical and chemical resistance [26]. A wide range of eco-friendly applications have already been envisioned for this novel bio-sourced material, ranging from the food, biomedical and pharmaceutical industries to textiles, electronics and filters/membranes [28]. When considering membrane applications, living filtration membranes have been studied, and interesting anti-fouling properties have been reported [29]. Attractively, the purification of this material (via the removal of living species, fermentation side-products and remaining tea and sugar) can generate cellulosic materials with high crystallinity and excellent mechanical properties, suitable for a larger range of applications [27]. The KBC material presents a highly porous 3D interconnected fibrous microstructure and a strong capacity to be functionalized with MOF crystals thanks to the high accessibility of abundant hydroxyl groups on its surface [30,31].

In the area of MOF materials, Zr-based UiO-66 is a well-known compound featuring triangular pores of ~6 Å, a very high surface area (1180–1240 m^2^·g^−1^ after heating at 300 °C) and unprecedented stability (resistance to most chemicals and upon washing in boiling water) [32]. Moreover, it offers attractive adsorption properties for gas separation (e.g., CO_2_/N_2_ and CO_2_/CH_4_ mixtures in harsh conditions) and water purification (heavy metal removal, oil/water separation, adsorption of dyes or antibiotics) [33,34].

The conjugation of this water-stable and biocompatible MOF with a hydrophilic cellulose support for water purification application has been only scarcely reported in the literature [15,35,36,37]. In a first example, a direct solvothermal method was used for the in situ growth of UiO-66 in the aligned lumen arrays of wood via the interaction of Zr^4+^ ions and the hydroxyl groups of cellulose forming the wood-cell walls [15]. The formed materials were used as filters for removing organic pollutants such as Rhodamine 6G (96% removal efficiency). The above approach is simple and uses a natural porous support, which can be shaped as a membrane for the dynamic filtration of waste water. However, the quantity of UiO-66 particles that can be grown in such wood filters (~5 mm thick) is limited by the cell wall surface (2.2 wt% of UiO-66 in [15]), and their location, distribution, aggregation degree and accessibility is hardly controlled. Other strategies using BC nanofibers could offer better control of these parameters, with excellent pore accessibility and higher UiO-66 loadings. However, most of the reported methods suffer from complex or multistep fabrication protocols such as seeding with MOF nanocrystals, mechanical fibrillation and fiber sieving, the addition of crosslinking agents, chemical modification of fibers, etc. As an example, UiO-66 nanocellulose aerogels were synthetized via a multistep strategy involving the dispersion of UiO-66 powder into a nanocellulose suspension (prepared via the microfibrillation of cellulose derived from wood), which was finally freeze-dried [35]. More recently, the uniform coverage of BC fibers with UiO-66 crystals (loading rate ~43%) was demonstrated thanks to a pre-coating of the former with polydopamine (PDA), favoring interactions with the terephthalic acid (BDC) ligand [36]. The resulting composite foam UiO-66/PDA/BC was tested for the adsorption of aspirin and tetracycline hydrochloride with removal efficiencies of ~75–77%.

In the present work, a natural KBC prepared via the processing of a tea–Kombucha SCOBY was used as a porous support for the in situ growth of UiO-66 crystals, applying a simple protocol without any deposition of crosslinking agent or chemical binder on the cellulose surface. UiO-66 nanoparticles were found to grow uniformly on the KBC–cellulose fibers via the interaction of Zr^4+^ ions with the abundant reactive hydroxyl groups of cellulose. Lightweight and flexible composite materials with hierarchical porosity were, thus, fabricated in a single step from a series of natural disk-shaped supports and without any chemical additives. Attractively, the KBC fibers in these innovative KBC-UiO-66 composite films (~0.5 mm thick) were entirely coated with uniform UiO-66 particles of ~100 nm in size (loading 15–20 wt%). The water cleaning effectiveness of such a composite material was tested by measuring the adsorption of various pollutants such as organic dyes including fluorescein (F), bromophenol blue (BPB) and Rhodamine 6G (Rh 6G).

## 2. Results and Discussion

As discussed in the introduction part of the manuscript, the strategy developed in this work consists of the fabrication of a cellulose-based material derived from Kombucha and its functionalization with MOF nanoparticles with a UiO-66 structure. This novel type of composite material was prepared via bacterial fermentation, growing Kombucha SCOBYs (membrane disks) at the air−water interface, before chemical cleaning, drying and their functionalization with MOFs synthetized in situ under solvothermal conditions. Such a synthesis method represents a simple procedure to generate strong interactions between MOFs and cellulose fibers. The physicochemical characteristics of the resulting composite material are described hereafter in relation to their morphology, composition and ability to remove model organic dyes from water media.

### 2.1. Physicochemical Characterization of the Materials

**Figure 1:**
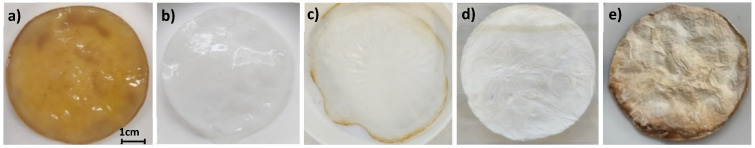
Kombucha SCOBY disk after (**a**) synthesis (KBC), (**b**) chemical treatment (KBC-CT), (**c**) conventional drying, (**d**) lyophilization (KBC-L) and (**e**) functionalization with MOF (KBC-UiO).

The different steps corresponding to the preparation of the final KBC-UiO composite (MOF-modified Kombucha-derived bacterial cellulose) are shown in Figure 1. It represents a Kombucha SCOBY disk after its synthesis (a), followed by chemical treatment (b), conventional drying (c) or lyophilization (d) and, finally, functionalization with MOF (UiO-66) particles (e).

**Figure 2:**
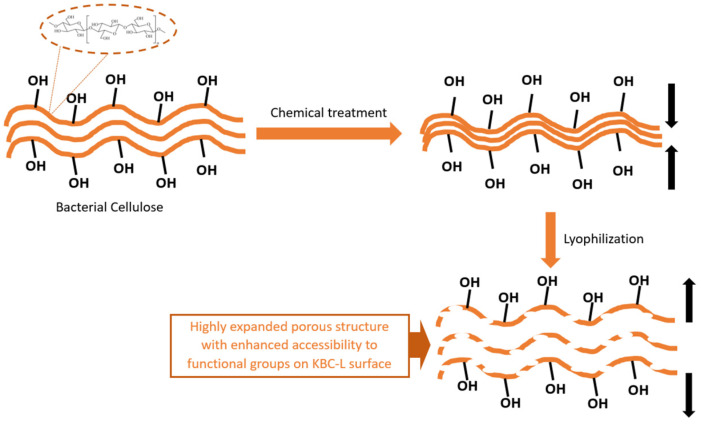
Schematic illustration of structural changes after chemical treatment and lyophilization of KBC.

It should be underlined that the chemical treatment was necessary to ensure the effective removal of sugars, yeasts, bacteria and other impurities present between the formed cellulose fibers/sheets, thereby blocking the porous network of the material. The chemical treatment/purification of the Kombucha SCOBY was carried out with NaOH and H_2_O_2_, as described elsewhere [38]. This protocol also enabled the efficient removal of the polyphenols and tannins inducing membrane disk discoloration (from brown to white), as observed in Figure 1b. The as-purified material was subsequently subjected to two different drying protocols (conventional drying at 80 °C for 12 h, Figure 1c and lyophilization, Figure 1d). This drying allowed to generate/increase the accessible active surface area and, thus, facilitate the subsequent incorporation of MOF particles (Figure 1e) as an active material for the removal of water pollutants. Standard Kombucha SCOBY is composed of numerous cellulose sheets/fibers (Figure S1), and, as schematically represented in Figure 2, both the chemical treatment and the lyophilization resulted in structural changes within the cellulose network.

Regarding the mechanical properties of KBC material, Young’s modulus values measured for the dried KBC-CT membrane (~2.4 GPa) were found to be similar to those measured for a commercial cellulosic membrane (Ultracel^®^ regenerated cellulose). Attractively, it should be noted that higher resistance to deformation was demonstrated for the KBC-CT sample (Figure S2).

**Figure 3:**
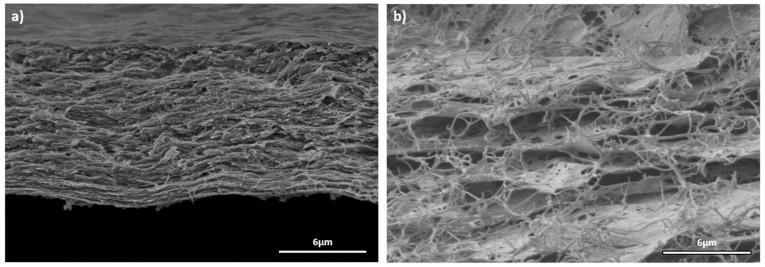
SEM images of (**a**) KBC-CT and (**b**) KBC-L.

The structural changes within the cellulose network due to the chemical and drying treatments were confirmed via SEM observations of KBC-CT and KBC-L. While the chemical treatment tends to approach the cellulose sheets closer to each other by removing impurities acting as spacers (Figure 3a), the lyophilization treatment widens the inter-layer distance leading to an expansion of the material porous structure (Figure 3b). The latter phenomenon is caused by the formation of water crystallites in chemically purified BC and their subsequent removal via sublimation, forming large macropores. As mentioned previously, such modification is particularly interesting for increasing the free volume in the BC host matrix to facilitate MOF incorporation. Indeed, the easy diffusion of reactive species within the expanded fibrous network is expected to facilitate access to cellulose functional groups, anchoring points for the growth of MOF crystals.

Water flux measurements were used to evaluate the permeability of the KBC membrane materials after the chemical and drying treatments. Our results are presented in Figure S4. Conventionally dried KBC membranes (without chemical treatment) were completely water-tight, as the bacteria, yeasts, sugars and other impurities were blocking the pores. On the contrary, the water flux for an undried KBC-CT sample could reach 78 L·m^−2^·h^−1^·bar^−1^ because its porous structure was free of organic residues (cleaned with NaOH/H_2_O_2_) and non-compacted. When this membrane was dried via conventional method, its porous structure collapsed, yielding compact membranes with extremely low water flux (below the detection limit of the experimental setup). Indeed, the interspaces between the cellulose sheets/fibers were too small and poorly interconnected (Figure 3a) to allow water transport through the membrane with a thickness of ~8 μm. The lyophization treatment of chemically treated membranes was an attractive option to stabilize an open porous structure with intermediate water permeability (37 L·m^−2^·h^−1^·bar^−1^). These results (Figure S4) are perfectly consistent with the expanded porous structure and interconnected macropores observed in Figure 3, enabling water transport through the membrane during the filtration process. When considering MOF growth, the lyophilization step is, thus, a relevant strategy to ensure the efficient infiltration of the reaction solution, allowing the uniform growth of MOF crystals within the porous structure of the KBC-L material.

**Figure 4:**
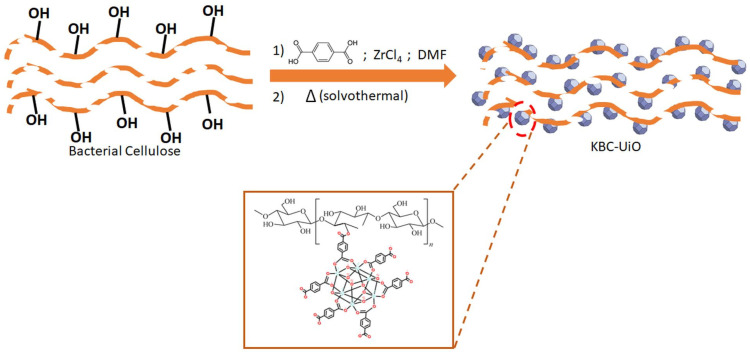
A schematic representation of the KBC-L surface modifications generated by the insertion of UiO-66 particles.

As described in the experimental part and schematically represented in Figure 4, the KBC-L porous structures were first impregnated with the UiO-66 precursor solution (composed of BDC as an organic ligand and ZrCl_4_ as a metal source in DMF). Then, the solution and KBC-L was transferred in a Teflon-lined stainless steel for the solvothermal treatment (120 °C, 24 h).

**Figure 5:**
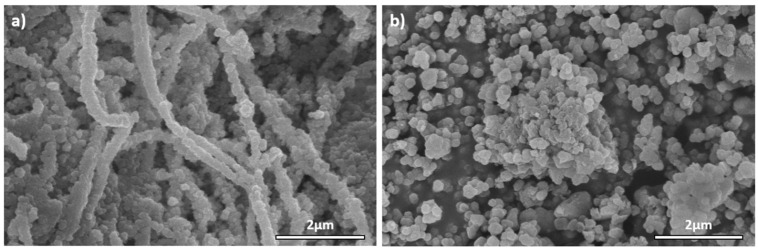
SEM images of (**a**) KBC-UiO sample and (**b**) unsupported UiO-66 particles.

SEM observation of KBC-UiO samples (Figure 5a) confirmed the presence of a large amount of MOF particles (UiO-66) particles grown on the fibers of the lyophilized cellulose structure. The particles were tightly stacked evenly covering the entire surface of the fibers. The crystal size was homogeneous (~100 nm) and slightly smaller than that measured in the unsupported UiO-66 powders prepared for comparison (Figure 5b).

It should be noted that the solvothermal treatment of KBC-L at 120 °C in the presence of UiO-66 precursors led to a slight color change in the cellulose (from white to brownish white (Figure 1e). This color modification did not have a negative impact on the integrity of the composite cellulosic material and did not damage its porous structure.

The as-prepared composite material should, thus, combine the advantages of a natural bio-sourced material with the exceptional adsorption properties of a MOF offering high accessibility to its adsorption sites.

**Figure 6:**
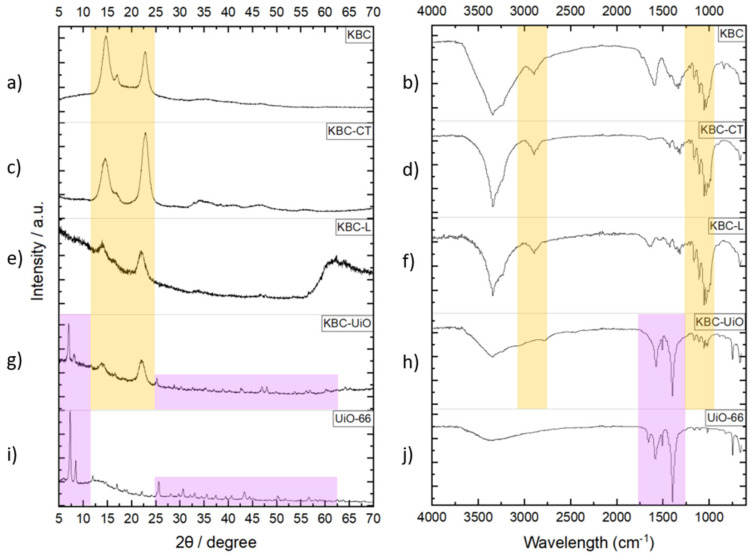
XRD patterns and IR spectra for the following: (**a**,**b**) pristine KBC; (**c**,**d**) KBC-CT; (**e**,**f**) KBC-L; (**g**,**h**) KBC-UiO; (**i**,**j**) unsupported UiO-66 powder.

XRD analysis of dried KBC and KBC-CT (Figure 6a,c) clearly evidenced the triclinic structure of bacterial cellulose, with similar high-intensity diffraction lines for both samples. Slight changes in the relative intensities of the peaks at 2θ = 15° and 23° were observed for KBC-CT due to the chemical treatment. On the other hand, the lyophilization step clearly led to a partial loss of crystallinity, as evidenced by the lower diffraction intensity (Figure 6e). Such amorphization of the material was attributed to harsh pressure and temperature variation conditions during the lyophilization step. As expected, the KBC-UiO composite material exhibited the XRD patterns of both cellulose and UiO-66 particles (Figure 6i), with very well-defined peaks at 2θ = 7° and 9° and in the range 15–70° [27,35]. Interestingly, a comparison of the XRD patterns for KBC-UiO and unsupported UiO-66 powders reveals differences in the intensity ratios of the (111) and (002) main diffraction peaks, which could be attributed to the effect of the cellulosic support on MOF crystal preferential orientation.

The IR spectra of the Kombucha SCOBY (Figure 6b) and its chemically treated counterpart (Figure 6d) present similarities, with well-defined peaks at ~3350 cm^−1^ and 1200–1400 cm^−1^ corresponding, respectively, to cellulose hydroxyl groups and residual impurities such as bacteria, yeasts, sugar and tea. The presence of numerous functional groups such as hydroxyls is important to favor the interaction of cellulose with the positive charge of metals [20], thus facilitating the in situ growth of MOFs on the cellulose fibers during the solvothermal synthesis step.

After chemical treatment, the vibration band at 1625 cm^−1^ corresponding to the C=O and OH functional groups of unreacted species within the cellulose structure disappeared, thus confirming the effective removal of impurities in KBC-CT. The IR spectra of KBC-UiO (Figure 6h) reveal the presence of the characteristic absorption bands of both UiO-66 and KBC. For the former, the FT-IR analysis (Figure 6j) confirms the presence of a C=O elongation band of the BDC ligand at 1660 cm^−1^ and several peaks at 1580 cm^−1^ and 1400 cm^−1^ corresponding to asymmetric and symmetric O–C–O elongation vibration bands. The absorption bands at ~670 cm^−1^, 745 cm^−1^ and 550 cm^−1^ correspond to the O–H vibration, C–H vibration and asymmetric elongation in Zr-(OC), respectively [27,35].

Thermogravimetric analyses of as-cultured KBC and its counterparts KBC-CT, KBC-L and KBC-UiO are shown in Figure S5. The TGA curve for the unsupported UiO-66 powder is also reported for comparison. The analysis of the KBC-UiO composite revealed a superposition of the thermal degradation of UiO-66 and KBC-L (significant weight losses of 10% at 525 °C and 36% at 325 °C for UiO-66 and KBC, respectively), thus suggesting similar stability compared to the individual materials. The total weight loss was found to reach approximately 85% at temperatures above 600 °C, which corresponds to the total degradation/carbonization of both KBC-L and UiO-66. Finally, it was confirmed that the quantity of UiO-66 within the cellulose support (15–20 wt%) estimated from TGA experiments (monitoring the MOF structure degradation) corresponds well to the gain in weight measured after the solvothermal growth of the MOF on the lyophilized cellulose support.

**Figure 7:**
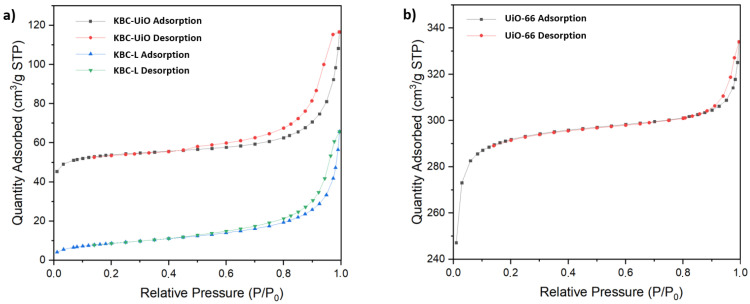
Nitrogen adsorption–desorption isotherms for (**a**) KBC-UiO and KBC-L and (**b**) UiO-66 unsupported powder.

Direct nitrogen sorption analysis on the KBC-UiO composite material revealed contributions of both type I (typical for microporous materials) and type IV (with hysteresis, typical for mesopores) isotherms (Figure 7a). The calculated specific surface area of the KBC-UiO composite material was S_BET_ = 180 m^2^·g^−1^. This value was attributed mainly to the presence of UiO-66, with a negligible contribution from the support itself. Indeed, the values of specific surface areas for unsupported UiO-66 powder and KBC-L were S_BET_ = 980 m^2^·g^−1^ and S_BET_ = 30 m^2^·g^−1^, respectively. The measured S_BET_ value for the composite material (180 m^2^·g^−1^) allowed to confirm the amount of UiO-66 within the cellulose support (estimated between 15 and 20 wt% via TGA and by mass gain after synthesis). Finally, the analysis of the adsorption isotherm in the low P/Po region enabled to evaluate a hydraulic pore diameter of 6.2 Å (modeless pore shape method—MP) for the UiO-66 MOF material.

### 2.2. Dyes Adsorption Performance—Effects of pH and Ionic Strength on Dye Adsorption

**Figure 8:**
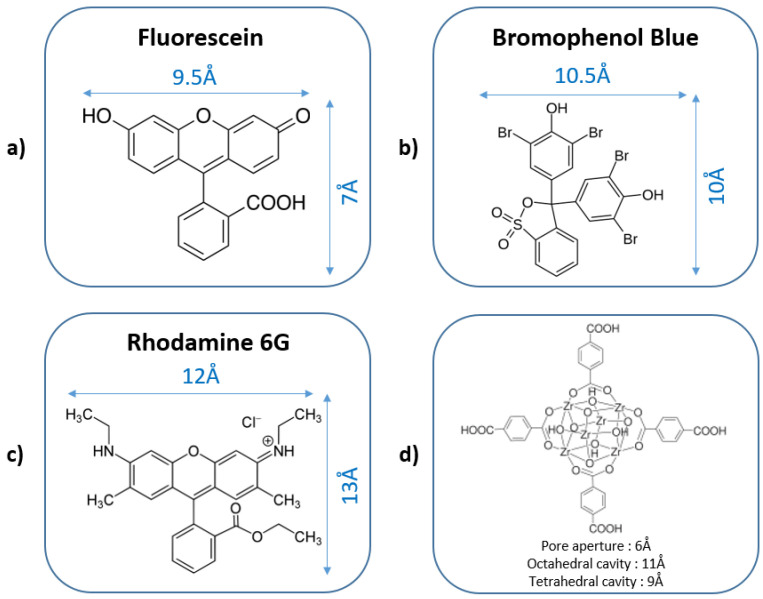
Schematic representations of the molecular structures and dimensions of (**a**) fluorescein, (**b**) bromophenol blue, (**c**) Rhodamine 6G and (**d**) UiO-66.

To evaluate the performance of the KBC-UiO composite material developed in this work, the effectiveness of water cleaning was tested by measuring the adsorption of three organic dyes: fluorescein (F), bromophenol blue (BPB) and Rhodamine 6G (Rh 6G). The molecular structures and dimensions of both the dye molecules and UiO-66 are shown in Figure 8. It is noteworthy that besides the molecular size of dye molecules, pH is also a critical factor known to significantly influence the parameters of sorption experiments. These parameters include the surface charge of the adsorbent, the degree of material ionization, the dissociation of functional groups at the active sites of the adsorbent and the structure of dye molecules. The experiments were carried out at pH 5 and pH 7 to reflect the mandatory regulations concerning the pH of the discharged water.

**Figure 9:**
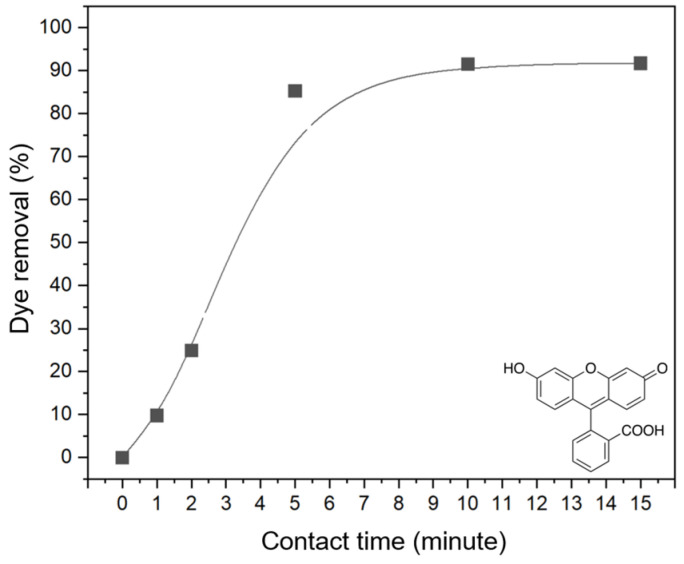
Adsorption kinetics for the KBC-UiO composite in contact with a fluorescein solution at pH = 5.

A preliminary series of experiments demonstrated that the adsorption kinetics for the KBC-UiO composite in contact with a fluorescein solution at pH = 5 typically follow the curve shown in Figure 9. From these measurements, it was concluded that the adsorption follows first-order kinetics, confirming that UiO-66 governs sorption via physisorption and surface diffusion mechanisms (Figure S6). It was found that the fluorescein removal efficiency reached a maximum value of ~90% after 10 min and then stabilized. Hence, a contact time of 10 min was chosen to compare the adsorption capacities for the other dyes and the effect of pH.

**Figure 10:**
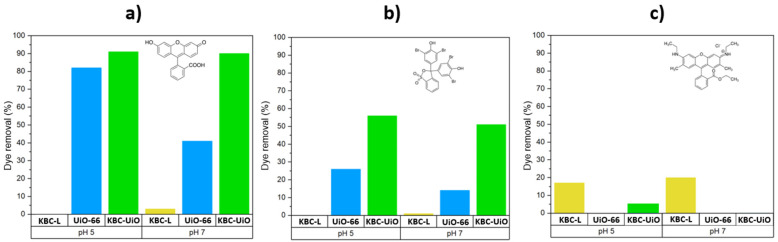
Comparison of dye removal efficiencies for the KBC-UiO composite, KBC-L sample and UiO-66 powder after 10 min of contact with (**a**) fluorescein, (**b**) bromophenol blue and (**c**) Rhodamine 6G dye solutions at pH 5 and pH 7.

A comparison of the adsorption capacity Q_t_ (amount of dye adsorbed on the adsorbent, mg·g^−1^) of the KBC-UiO samples after 10 min of contact (static conditions) with the dye solutions at pH 5 and 7 is presented on Table 1 and Figure 10. The performances for equal quantities of the individual components KBC-L and UiO-66 are also reported for comparison. It clearly appears that the unsupported UiO-66 powder is not at all effective for the adsorption of Rhodamine 6G (Rh 6G) at these pH values. Indeed, the surface charge interactions are not favorable, and the Rh 6G molecule is too large to penetrate the UiO-66 cavities. On the other hand, Rh 6G can enter the KBC-L fibrous macrostructure and become trapped on/in the cellulose fibers. Thus, the pure cellulose sample (KBC-L), with its numerous functional groups present on the surface, is capable of adsorbing more than 2.2 mg·g^-1^ of Rh 6G under similar conditions. As a consequence, the KBC-UiO composite is not effective for the adsorption of Rh 6G compared to pure KBC-L. Given these results, it is surprising that Guo et al. [15] claim a high removal efficiency of Rh 6G (97%) after filtration on a wood structure containing 2.2 wt% of UiO-66 particles. It is unclear whether this performance is linked to the MOF or the wood itself, which is rich in cellulose. In good agreement with our results, Jia et al. [39] also reported that for MOF-808 (another Zr-based MOF), the adsorption of fluorescein is facilitated compared to Rhodamine (despite the electrostatic interactions and μ-OH and π–π stacking for both dyes) because only fluorescein can potentially penetrate the pores. Finally, the material performance was compared with other cellulose MOF-based adsorption systems reported in the literature (Table S1), thus confirming its competitive adsorption capacity.

Concerning the adsorption of fluorescein and bromophenol blue, a synergistic effect between KBC and the UiO-66 crystals uniformly covering the cellulose fibers is highlighted in Figure 10 and Table 1. Indeed, for these two dyes and whatever the pH value considered (pH 5 or pH 7), the KBC-UiO composite sample always offers a greater adsorption capacity than the simple sum of the performance of its individual constituents (equivalent weight). For example, at pH 7, the adsorption capacity of the KBC-UiO composite reaches a value of 9.7 mg·g^−1^ for fluorescein (removal efficiency of 90%), i.e., twice as high as the sum of the performance for KBC-L and UiO-66 powder. In the same way, for bromophenol blue at pH 7, the adsorption capacity of KBC-UiO reached a value of 15 mg.g^−1^ (removal efficiency of 55%), i.e., more than three times greater than the sum of the performance for KBC-L and the UiO-66 powder. The same trend is also observed to a lesser extent at pH 5 for both dyes. Taking into account the low adsorption capacity of KBC-L, the measured performance essentially depends on the specific characteristics and accessibility of the UiO-66 crystals. The pristine KBC was confirmed to exhibit virtually zero adsorption efficiency, while gradually increasing the UiO loading in the KBC-L structure resulted in a linear increase in the adsorption capacity. The maximum value was reached for the composite material containing ~15 to 20 wt% UiO-66. We found that higher UiO loadings resulted in a partial loss of mechanical stability. The superior efficiency of the composite, thus, demonstrates the interest in using KBC-L as a support to grow uniform crystals of UiO-66 on its cellulose fibers. Consequently, such a derived hierarchical structure is effective at producing materials that are easy to implement in processes targeting the adsorption of dyes from the water environment.

Between pH 5 and pH 7, cellulose and UiO-66 have different zeta potential values: negative at pH 5 and positive at pH 7 [21,40]. The adsorption of HO^−^ ions on the surface of the adsorbent increases its negative charge. In this pH range, fluorescein is in its anionic form [39], essentially mono-anionic at pH 5 and 50% di-anionic at pH 7. This explains why the quantity of fluorescein adsorbed on the UiO-66 powder was higher at pH 5 compared to pH 7. Interestingly, this effect was not clearly observed for the KBC-UiO composite material, and similar adsorption efficiencies were obtained at pH 5 and pH 7.

Bromophenol blue also occurs in its anionic form between pH 5 and 7 [41], but its molecular size (10 × 10.5 Å) is larger than that of fluorescein (Figure 8b). Indeed, the size of the fluorescein molecule (7 × 9.5 Å) is close to those of the pore openings in UiO-66, and the molecules could, thus, potentially penetrate into the cavities (Figure 8a). Such a molecular size effect, coupled with charge effects, could explain the lower removal efficiency of bromophenol blue compared to fluorescein. Indeed, the electrostatic interactions and hydrogen bonds between the SO_3_H group of bromophenol blue and the –OH groups of the UiO-66 cages are not sufficient to explain the differences in removal efficiency for the two dyes. To sum up, in all cases after just 10 min of contact with an anionic dye solution (pH between 5 and 7), the KBC-UiO composite material is capable of removing up to 90% of the dye in the case of fluorescein, as well as more than 50% in the case of bromophenol blue. A combination of surface charge, hydrogen bonding and steric hindrance effects (the ease of the penetration of the dye into the MOF structure) are the claimed mechanisms explaining the difference in removal efficiency between these two dyes. On the other hand, the KBC-UiO composite is not effective for the adsorption of a large cationic dye such as Rhodamine 6G (12 × 13 Å). In fact, the pure KBC-L material, with its expanded fibrous structure and numerous surface –OH groups, is found to actually be more effective, although offering limited performance. To complete this study, material regeneration experiments are carried out to evaluate the reuse of KBC-UiO for repetitive adsorptions of organic dyes. The best results are obtained by using DMF as the regenerating solvent (~6 mol·L^−1^), leading to a recovery of 70–50% depending on the number of adsorption/regeneration cycles, as shown in Figure S7. A stabilization of performance at ~50% dye removal efficiency is observed after three regenerations, likely due to the presence of fluorescein blocked in the UiO porous framework. In fact, the mild regeneration conditions applied (orbital shaker-160 rpm, 1 h, 25 °C) can hardly extract the species blocked in the internal pores of the UiO structure. Once these adsorption sites are blocked, the next regeneration/sorption cycles only concern external crystalline surfaces; however, the abatement performance still remains very attractive.

Given the demonstrated effectiveness of the KBC-UiO composite material for the removal of anionic dyes such as fluorescein after only 10 min in static conditions, the next steps will focus on the application of the promising KBC-UiO composite material in dynamic conditions for water filtration via the membrane process. Further research will be conducted on the influence/optimization of KBC-L membrane thickness. Moreover, the long-term stability in the presence of mixtures of dyes will be the subject of our work.

## 3. Materials and Methods

### 3.1. Formation of KBC Membranes

Kombucha is a traditional fermented tea drink, obtained from a complex culture of acetic acid bacteria (Acetobacter, Gluconobacter and Komagataeibacter), yeasts (Saccharomyces cerevisiae, Brettanomyces bruxellensis, Schizosaccharomyces pombe and Zygosaccharomyces rouxii) and, often, lactic acid bacteria (Lactobacillus, Lactococcus) [38]. The symbiotic culture initiates the fermentation of tea and sugars, producing a floating biofilm (membrane) over several days/weeks. In this work, the KBC membranes were produced from a natural “mother” SCOBY using a conventional method [42,43,44,45] in 3 steps: (i) the growth of Kombucha biofilms, (ii) chemical cleaning treatment and (iii) controlled drying.

#### 3.1.1. Growth of Kombucha Biofilms

Kombucha biofilms were grown from a “mother” SCOBY provided by a Kombucha brewery (BB Kombucha-France [46]). The SCOBY was placed in a growth solution (250 mL) consisting of anhydrous glucose (17.5 g; ≥99.5%, Sigma-Aldrich), 250 mL of a tea infusion and a small quantity of a previous Kombucha brew (15 mL). The tea infusion was previously prepared by steeping 1 g of black tea (Lipton Yellow Label, filter paper bag) for 15 min in 250 mL of deionized (DI) water heated to 100 °C. The mixture was stored in a temperature-controlled room at 25 °C, and the SCOBY grew at the liquid–air interface until reaching a thickness of 2 to 3 mm in 14 days. The diameter of the SCOBY corresponded to the beaker diameter, i.e., typically 6.5 cm. After 14 days, the SCOBY “daughter” was recovered, washed with DI water and chemically treated to remove living species (bacteria and yeasts) and the remaining SCOBY feed (tea and sugar).

#### 3.1.2. Chemical Cleaning Treatment of the SCOBY

After the DI water cleaning step, SCOBY daughters were immersed in a freshly prepared NaOH solution (1 mol·L^−1^; 98.5%, ChemLab, Zedelgem, Belgium) for 1 h, with the operation being carried out twice. Finally, they were placed in a diluted hydrogen peroxide (1.5 wt%; 35%, ChemLab) solution for 2 h. The as-obtained SCOBY disk had an average pore size of 30 nm, as reported in the literature [43]. After the chemical treatment, the derived cellulosic membranes (KBC-CT) were dried in two different ways to remove all water from the material.

#### 3.1.3. Drying/Stabilization of Cellulosic Membranes

The KBC-CT membranes were dried either at 80 °C for 12 h in an oven (conventional drying) or via a freeze-drying process (lyophilization). In the latter case, the KBC-CT membranes were first placed in a freezer for 12 h and then in the freeze-dryer (Cryotec Cosmos, Lunel Viel, France) for 6 h. The as-obtained membrane was denominated KBC-L.

### 3.2. Formation of KBC-UiO Composite Membranes

To grow and anchor UiO-66 nanoparticles in situ on the cellulose fibers of the KBC-L membrane, the latter was first impregnated under vacuum with a reaction solution prepared using a method reported elsewhere for the synthesis of UiO-66 powders [40]. A standard solution was prepared by dissolving ZrCl_4_ (314 mg, 2.7 mmol, 98%, ThermoFischer Scientific, Waltham, MA, USA) in 15 mL of N,N-dimethylformamide (DMF, 99.8%, Sigma Aldrich, Saint-Louis, MI, USA). In a separate beaker, 1,4-benzenedicarboxylic ligand (BDC, 224 mg, 2.7 mmol, 98%, Alfa Aesar, Haverhill, MA, USA) was dissolved in 15 mL of DMF. Both solutions were stirred until a complete dissolution of the solid materials and the metal precursor solution was then added dropwise to the ligand solution. Subsequently, the KBC-L membrane was immersed for impregnation by the as-prepared reaction solution for 10 min under primary vacuum. The impregnated KBC-L membrane together with the ligand–metal precursor solution were then introduced into a Teflon-lined stainless steel autoclave and heated at 120 °C for 24 h. After the reaction, the KBC-UiO composite material was recovered and washed in a 50 mL DMF bath for 30 min before its immersion in a deionized water bath for 1 h to remove all impurities and unreacted ligands. Finally, the as-obtained KBC-UiO was dried in a conventional oven at 80 °C overnight [24]. For comparison, UiO-66 powder was also prepared using the same solutions and protocols described above (but without the KBC-L support) [40].

### 3.3. Physicochemical Characterization of Materials

A high-accuracy digital microscope (Keyence VHX-7000, Bois-Colombes, France) was used for initial observations of KBC samples. A Scanning Electron Microscope (FESEM, Hitachi S-4800, Tokyo, Japan) was then applied to observe the morphology of KBC membranes after chemical/drying treatments and the growth of UiO-66 material. Before SEM observations, the samples were cut into small pieces (5 mm squares) and sputter-coated with platinum.

Gas physisorption data were collected using a Micromeritics ASAP-2020, Norcross, GA, USA at 77 K, measuring N_2_ adsorption–desorption isotherms. Samples were previously out-gassed for 12 h at 200 °C under vacuum. The specific surface area was determined by using the standard Brunauer–Emmett–Teller (BET) equation.

X-ray Diffraction (XRD) patterns were recorded using a PANalytical X’pert MDP-Pro, New York, NY, USA, equipped with an X-ray detector with CuKα radiation (λ = 1.54 Å) at a 40 kV voltage and a 15 mA current. Measurements were performed at ambient temperature with a scan speed of 0.027° min^−1^, a step size of 0.017 and a 2θ range of 5–70°.

Infrared spectroscopy analysis was performed on a Nexus FT-IR with a diamond Attenuated Total Reflectance (ATR). Samples were scanned 32 times, and spectra were recorded with wavelengths from 4000 to 400 cm^−1^.

Thermogravimetric analysis (TGA) was performed using a SDT Q600 from TA Instruments and platinum crucibles. Thermograms were recorded at a heating rate of 10 °C·min^−1^ between 25 and 1000 °C with a flow of nitrogen at 20 mL·min^−1^.

The mechanical properties of the pristine KBC and KBC-CT membranes (both conventionally dried) were compared to those of a commercial cellulosic membrane (Ultracel^®^ regenerated cellulose) using an Instron 3366 apparatus and Bluehill 3 software. Tests were carried out with a 10 kN cell at a speed of 5 mm·min^−1^ and in compliance with the ISO 527-3 standard.

In order to evaluate the evolution of the material structure after chemical and drying treatments, the pure water permeability of the membrane materials was measured at room temperature using a dead-end ultrafiltration cell (Amicon^®^ 50 mL cell, Millipore Sigma, Saint-Louis, MI, USA) with an effective membrane area of 13.4 cm^2^. Permeation tests were carried out at increasing transmembrane pressures up to 3 bars, and the permeate was collected continuously in a vessel located on a digital balance to measure the evolution of its flow (Figure S3).

### 3.4. Dye Adsorption Performance

The performances of the KBC-UiO samples for organic dye abatement were evaluated with 3 model molecules (purchased from Sigma Aldrich) featuring different ionic behaviors (depending on pH): bromophenol blue (neutral/anionic), Rhodamine 6G (cationic/neutral) and fluorescein (neutral/anionic). Dye solutions (5 ppm) were prepared in DI water at pH ~5 and 7 to simulate realistic polluted water. Solutions (0.1 mol·L^−1^) of hydrochloric acid (37%, ChemLab) and sodium hydroxide (98.5%, ChemLab) were used for adjusting the pH.

KBC-UiO samples (10 mg) were added into small beakers (10 mL) filled with 2 mL of dye solution (5 ppm).

In parallel for the sake of comparison, the performances of individual pristine KBC and UiO-66 powders were also evaluated using the quantities of KBC and UiO-66 corresponding exactly to those in the composite material. All these experiments were performed in static conditions.

The concentrations of the dye solutions before and after the adsorption experiments were determined using a UV-Vis spectrophotometer (Jenway 7315 spectrophotometer) at the maximum adsorption band of each dye at the corresponding pH. All concentration measurements were repeated three times.

The removal efficiency was calculated using the following equation:

%Removal = [(C_t_ − C_o_)/C_o_] × 100% (1)

where C_o_ (mg·L^−1^) and C_t_ (mg·L^−1^) are the dye concentrations at initial time and time t, respectively.

During the experiment, 0.4 mL samples were taken periodically to determine the remaining dye concentration in the solution until reaching a steady value.

The adsorption capacity (Q_t_) was calculated as follows:

Qt = (C_o_ − C_t_) V/m (2)

where Q_t_ is the adsorption capacity at a given time t (mg·g^−1^), m is adsorbent weight (g) and V is dye solution volume (L).

## 4. Conclusions

In this work, we describe for the first time an original approach for the functionalization of Kombucha (KBC)-derived natural bacterial cellulose with UiO-66 particles homogeneously covering the cellulose fibers. This strategy for the modification of such a bio-sourced support results in an innovative flexible composite material with great potential in environmental remediation, notably for the removal of toxic organic dye pollutants from industrial waste waters. Moreover, thanks to the biocompatibility, attractive textural characteristics, surface properties, and stability in water of both UiO-66 and KBC, the present system represents a promising alternative candidate to other adsorbents and synthetic cellulose derived from industrial polymers. The synergistic effects involving the attractive adsorbent properties of UiO-66 and the surface chemistry of this natural KBC were shown to be of great benefit for the adsorption of organic pollutants, especially anionic dyes. Indeed, promising adsorption properties were evidenced for fluorescein or bromophenol blue in water at pH 5 and pH 7 (more than 90% and 50% removal efficiency, respectively, after 10 min in static conditions). As a conclusion, the results of this study pave the way for the design of new effective bio-sourced advanced materials applicable in the area of hybrid adsorption–filtration membrane systems for the treatment of contaminated water [41]. The composite filters, offering high adsorption efficiencies and short contact times, could be effectively used in water filtration to remove traces of organic pollutants such as volatile organic compounds, iodine- and phenolic-based compounds, drugs, pesticides and toxins. The elimination of heavy metal ions (e.g., Cr^3+^, Cu^2+^, Co^2+^, Ni^2+^, Zn^2+^, etc.) and toxic elements such as arsenic ions is also an interesting specificity of UiO-66-based adsorbents [47,48]. To further improve the material performance, better control of the structural modification of the cellulose during the lyophilization step, the judicious selection of MOF adsorbents and their optimized loading into the cellulose fibrous matrix are currently being considered.

## Figures and Tables

**Table 1:** Adsorption capacities (Qt) of the KBC-UiO composite, KBC-L sample and UiO-66 powder after 10 min of contact with fluorescein, bromophenol blue and Rhodamine 6G dye solutions at pH 5 and pH 7.

	Qt (mg·g^−1^)								
	Fluorescein			Bromophenol Blue			Rhodamine 6G		
	KBC-L	UiO-66	KBC-UiO	KBC-L	UiO-66	KBC-UiO	KBC-L	UiO-66	KBC-UiO
**pH 5**	0	8.4	9.4	0	7.3	15.9	2.0	0	0.6
**pH 7**	0.3	4.5	9.7	0.2	4.3	15.0	2.4	0	0

## Data Availability

The data presented in this study are available on request from the corresponding author. The data are not publicly available due to the institution’s policy.

## Supplementary Materials

The following supporting information can be downloaded at https://www.mdpi.com/article/10.3390/molecules29133057/s1. Figure S1: Optical microscope images of KBC (after conventional drying at 80 °C for 12 h; Figure S2: Stress–strain curves for dried KBC, KBC-CT and a commercial cellulosic membrane (Ultracel^®^ regenerated cellulose); Figure S3: Water permeation setup and Amicon^®^ 50 mL cell for membrane disks (Millipore Sigma); Figure S4: Pure water flux measured versus transmembrane pressure through undried KBC-CT and conventionally dried KBC and KBC-CT in comparison with lyophilized KBC (noted KBC-L); Figure S5: TGA curves for the conventionally dried KBC and KBC-CT, for the lyophilized KBC-L and for the KBC-UiO composite. The TGA curve for dried UiO-66 powder is reported for comparison; Figure S6: Adsorption plot (first-order kinetic model) of fluorescein by KBC-UiO; Table S1: Comparison of the KBC-UiO performance with other cellulose/MOF-based adsorption systems for the dye removal reported in the literature; Figure S7: Fluorescein removal rate using pristine (1) and reused KBC-UiO after consecutive regeneration cycles (2–5) with an orbital shaker (160 rpm, 1 h, 25 °C). Equation (S1): first-order model. Equation (S2): equation after integration. References [15,35,49,50,51,52,53] are from Supplementary Materials.
