# Supplementary material for: Synergistic Effect of UiO-66 Directly Grown on Kombucha-Derived Bacterial Cellulose for Dye Removal
Source: Molecules. 2024 Jun 27;29(13):3057. doi: 10.3390/molecules29133057 (PMC11243549; doi:10.3390/molecules29133057)
Supplement: Supplementary file 1 [file molecules-29-03057-s001.zip › molecules-3040034-supplementary.pdf]

SUPPLEMENTARY INFORMATION

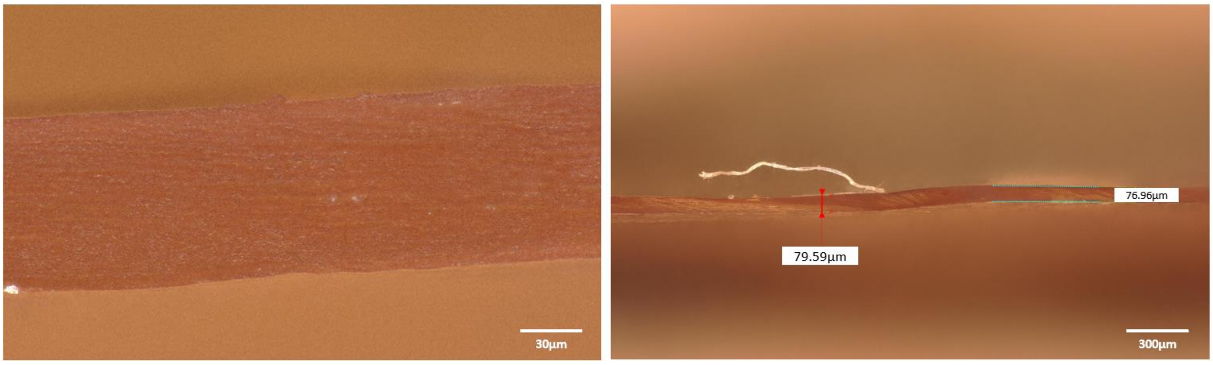

Figure S1: Optical microscope images of KBC (after conventional drying at 80°C for 12 hours).

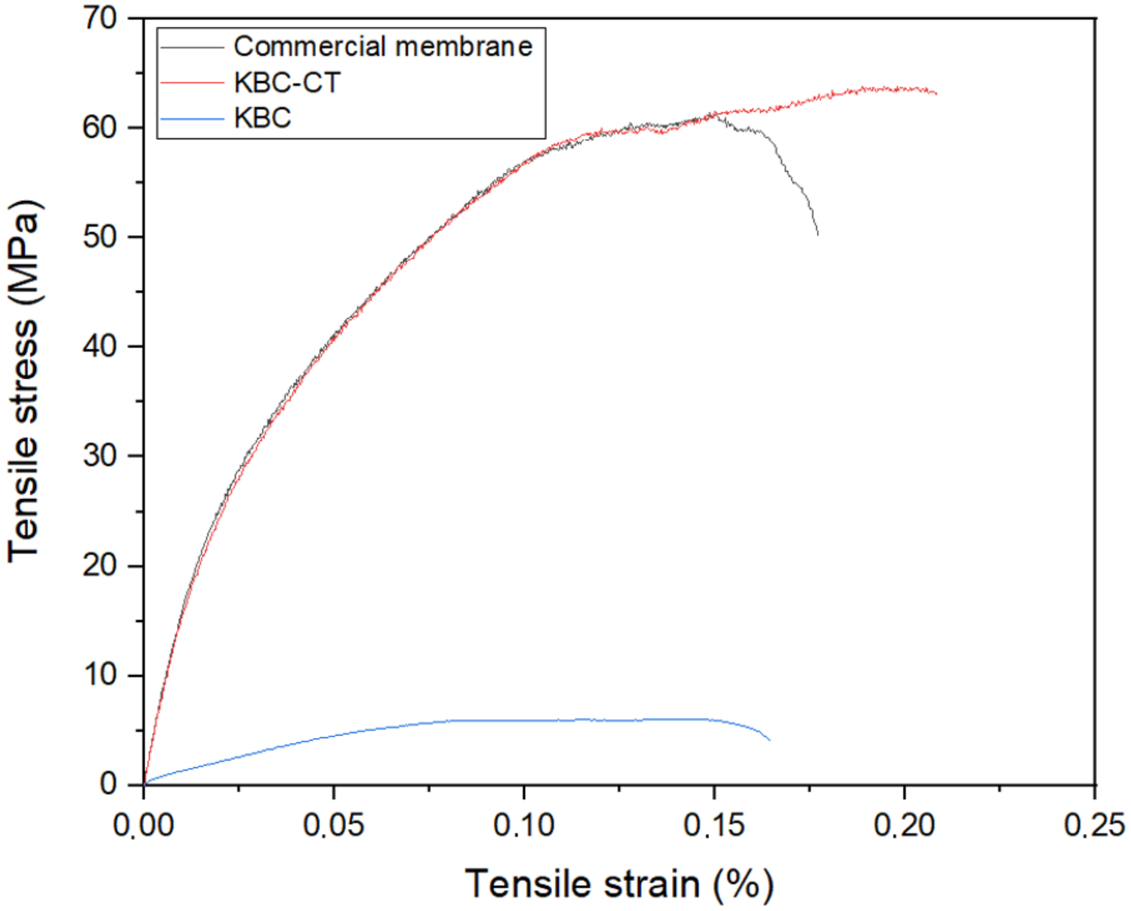

Figure S2: Stress-strain curves for dried KBC, KBC-CT and a commercial cellulosic membrane (Ultracel® regenerated cellulose).

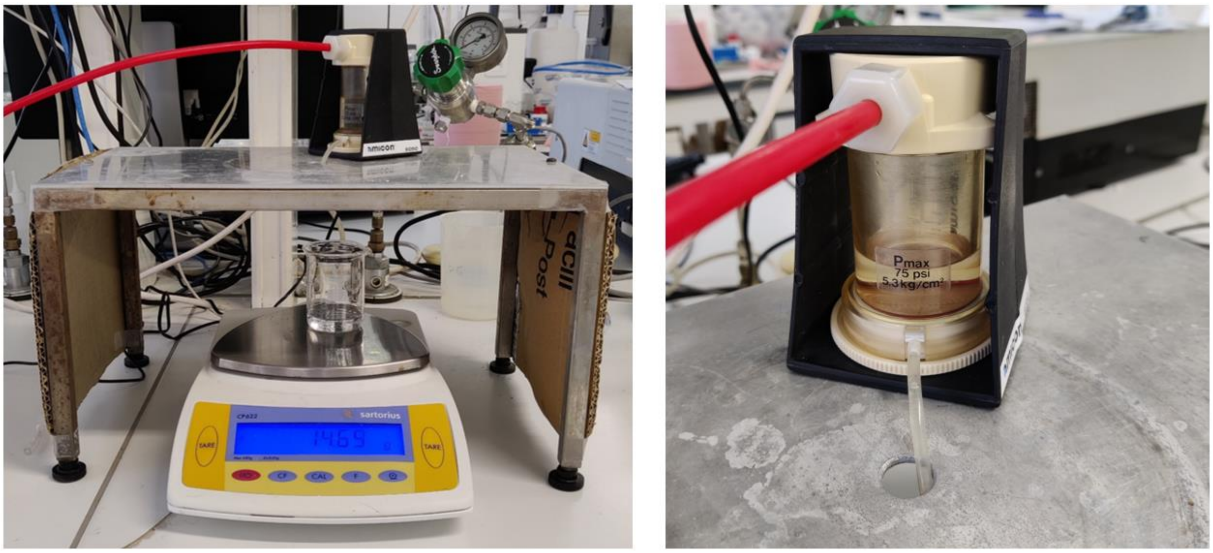

Figure S3: Water permeation setup and Amicon® 50 mL cell for membrane discs (Millipore Sigma).

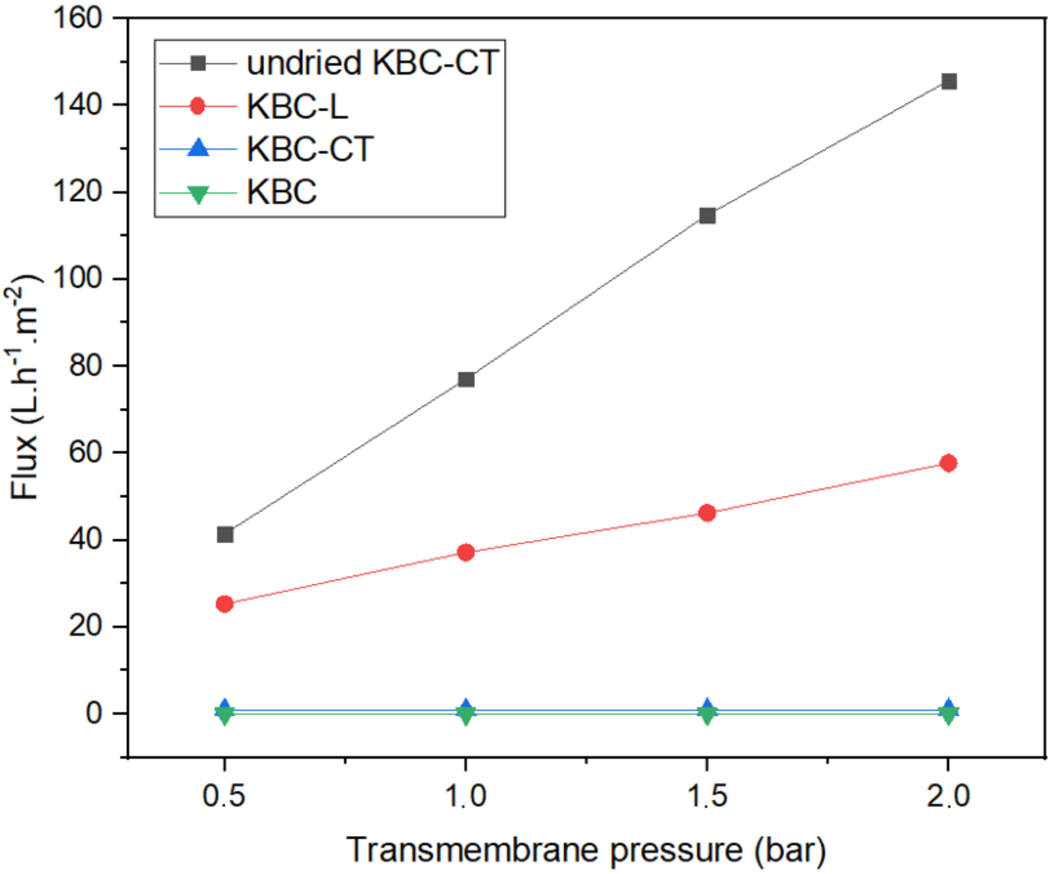

Figure S4: Pure water flux measured versus transmembrane pressure through undried KBC-CT and conventionally dried KBC and KBC-CT in comparison with lyophilized KBC (noted KBC-L).

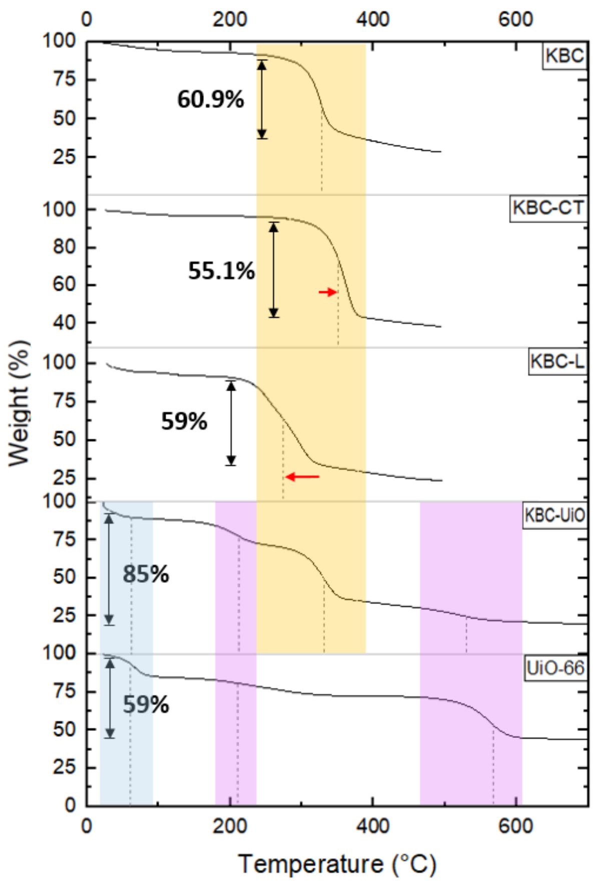

Figure S5: TGA curves for the conventionally dried KBC and KBC-CT, for the lyophilized KBC-L, and for the KBC-UiO composite. The TGA curve for dried UiO-66 powder is reported for comparison.

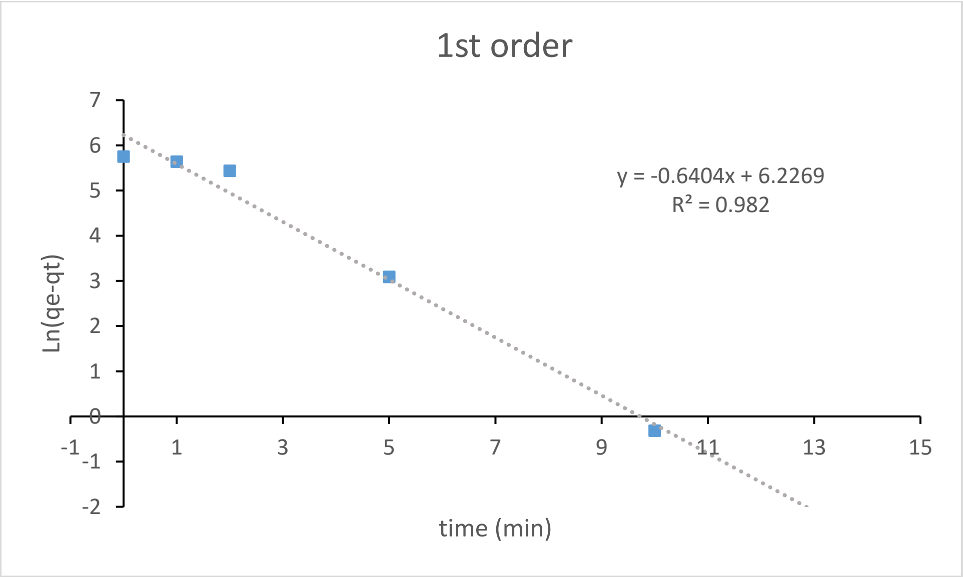

Figure S6: Adsorption plot (first-order kinetic model) of Fluorescein by KBC-UiO

Different kinetic models were used to elucidate the adsorption mechanism of fluorescein on KBC-UiO and to determine the rate dominant step of the adsorption process. The best fit was obtained with the

first-order kinetics model providing adsorption rate constant k_1=-0.640 min^{-1} with the coefficient of determination R^2 = 0.982 [52,53].

The first-order model could be described as follows:

$$q_t = q_e(1 - e^{k_1 t}) \qquad (S1)$$

In which q_e and q_t represent the quantity of fluorescein adsorbed at equilibrium, t the adsorption time, and K_1 the rate constant of the first-order reaction.

After integration, Equation (S1) can be expressed as:

$$\ln(q_e - q_t) = ln(q_e) - k_1 t \qquad (S2)$$

Table S1: Comparison of the KBC-UiO performance with other cellulose/MOF-based adsorption systems for the dye removal reported in the literature. (* number of tested cycles)

**Material**	**Support**	**MOF loading (wt%)**	**Dye**	**Maximum removal efficiency (%)**	**Contact time (min)**	**Dye solution pH**	**Regenerability (Cycles) ***	**Ref.**
**ZIF-8/CNC**	Cellulose nanocrystals	50	Malachite green	97	240	-	3	https://doi.org/10.1016/j.jcis.2020.03.076 [49]
**HKUST-1/OCBs**	Cellulose from Corncobs	-	Methyl orange	55	60	5	4	https://doi.org/10.1016/j.carbpol.2019.115042 [50]
**ZIF-67/CA**	Cellulose Aerogel	41	Methyl orange	90	50	-	5	https://doi.org/10.1007/s10570-019-02883-2 [51]
**UiO-66/Wood**	Cellulose from Wood	2	Rhodamine 6G	96	5	1 - 7 - 14	6	https://doi.org/10.1021/acs.est.8b06564 [15]
**UiO-66/nanocellulose**	Cellulose Aerogel	50	Methyl orange /Methylene blue	92	200	7	4	https://doi.org/10.1016/j.cej.2019.04.022 [35]
**UiO-66/KBC**	Kombucha bacterial cellulose	15	Fluorescein	~90	10	5 - 7	5	This work

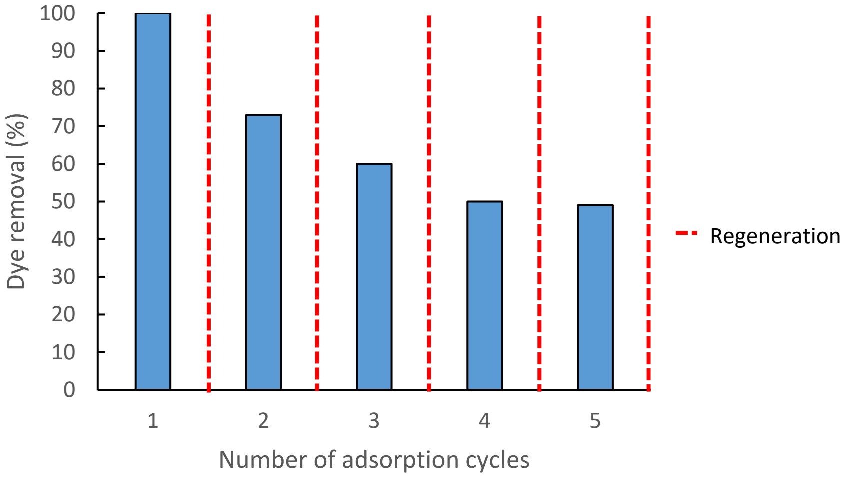

Figure S7: Fluorescein removal rate by pristine (1) and reused KBC-UiO after consecutive regeneration cycles (2-5) with an orbital shaker (160 rpm, 1h, 25°C).
